# Leukocytosis and Expression of Bovine Leukemia Virus microRNAs in Cattle

**DOI:** 10.3389/fvets.2020.00272

**Published:** 2020-06-09

**Authors:** Gleb Yu. Kosovsky, Valery I. Glazko, Galina V. Glazko, Boris L. Zybaylov, Tatiana T. Glazko

**Affiliations:** ^1^FSBEI HPE Russian State Agrarian University - Moscow Timiryazev Agricultural Academy, Moscow, Russia; ^2^FSBSI V.A. Afanasyev RI for Fur and Rabbit Farming, Moscow, Russia; ^3^Department of Biomedical Informatics, University of Arkansas for Medical Sciences, Little Rock, AR, United States; ^4^Department of Biochemistry and Molecular Biology, University of Arkansas for Medical Sciences, Little Rock, AR, United States

**Keywords:** microRNA, Bovine Leukemia Virus (BLV), retroviruses, farm animals, leukocytosis

## Abstract

Bovine Leukemia Virus (BLV) is an established model for studying retroviral infections, in particular the infection by the human T-cell leukemia type 1 (HTLV-1) virus. Here, we quantified gene expression of several BLV-related genes: effector protein of *T* and NK-killer cells NK-lysin (*Nklys*), reverse BLV transcriptase *pol, BLV* receptor (*blvr)*, and also key enzymes of the microRNA maturation, Dicer (*dc1*) and Argonaut (*ago2*). The differences in the expression of the above genes were compared between five groups: (1) BLV infected cows with high and (2) low lymphocyte count, (3) with and (4) without BLV microRNA expressions, and (5) cows without BLV infections (control group). As compared to control, infected cows with high lymphocyte count and BLV microRNA expression had significantly decreased *Nklys* gene expression and increased *dc1* and *ago2* gene expressions. Few infected animals without *pol* gene expression nevertheless transcribed BLV microRNA, while others with *pol* gene expression didn't transcribe BLV microRNA. Notably, *Pol* expression significantly (*P* < 0.05) correlated with *dc1* expression. For infected animals, there were no direct correlations between the number of leukocytes and *pol, Nklys*, and BLV microRNA gene expressions. *Blvr* gene expression is typical for juvenile lymphocytes and decreases during terminal differentiation. Our data suggest that BLV infects primarily juvenile lymphocytes, which further divide into two groups. One group expresses BLV DNA and another one expressed BLV microRNA that decreases host immune response against cells, expressing BLV proteins. It is suspected that regulatory microRNAs play a significant role in the bovine leukemia infections, yet the precise mechanisms and targets of the microRNAs remain poorly defined. Vaccines that are currently in use have a low response rate. Understanding of microRNA regulatory mechanisms and targets would allow to develop more effective vaccines for retroviral infections.

## Introduction

Retroviruses are the main source of infections in humans and farm animals (1). During the course of evolution, retroviruses acquired the ability to suppress host immunity. Interestingly, the main players in the suppression process are viral microRNAs. They modify host metabolic pathways not only by affecting mRNA translation but also by interacting with host microRNA profiles (2). Viral microRNAs also interfere with regulation of host microRNAs that control the processes of cell division and innate immunity (3–5).

The ever-growing popularity of microRNA as a research target rests on its regulatory role in transcriptomic and epigenomic processes (6). The epigenome encompasses epigenetic marks such as DNA methylation, histone modifications, chromatin remodeling, and other molecules that can transmit epigenetic information such as non-coding RNAs, in particular microRNAs (7).

There are more than 1,600 structural genes, involved in a variety of metabolic pathways, including those associated with the immune system, that are regulated in a different way in present-day high-productive cattle breeds as compared to ancient ancestral forms (8). The regulatory differences are related to different targets of microRNA activity (8). Many microRNAs, participating in milk protein metabolism and quality, as well as regulating immune system at various stages of cow lactation, are now identified (9, 10).

Recently, microRNAs of Bovine Leukemia Virus (BLV), namely their organization, expression, and targets, became a focus of a large number of research projects, partially because BLV is closely related and similar to a Human T-cell leukemia virus Type I and II (HTLV-I and II) (1, 11–13). BLV-induced infection has two stages: proviral BLV DNA insertion into B-lymphocytes of host genome and proliferation of infected B-cell clones with increased leukosogenic potential (14). It was suggested that those clones do not express BLV proteins, but instead actively express microRNAs, influencing lympholeukosis. Sequencing of the small RNAs isolated from B-lymphomas of sheep, infected with BLV, revealed 10 regions 20–23 bp each of five BLV microRNAs that were transcribed from proviral DNA (from 6,398 to 6,906 bp) between the *env* gene and *2 R3* exon (12). It was found that in lymphoma cells, BLV microRNA transcripts represent 40% of all cellular microRNAs and their transcription involves RNA polymerase III. While 5′LTR hypermethylation is associated with BLV repression, BLV micro-RNA cluster remains active (13). The authors assume that transcriptional activation of BLV micro-RNA cluster in primary tumors and pre-leukemic clones is caused by negative selection against cell clones expressing BLV proteins provided by host immune system. Moreover, it was shown that expression of the one of BLV microRNAs, BLV-miR-B4, that has the same seed nucleotide sequence (2–7 nucleotides) as miR-29 from cattle genome, is greater than expression of miR-29 – a member of miR-17-92 family (oncomir-1) (3, 4). Overexpression of miR-29 was found in BLV-infected tumor cells, as well as in B lymphomas of human and mice (5). It is also known that BLV microRNAs play important role in BLV-induced leukoses, competing, with the help of RNA polymerase II, with antisense transcript of proviral DNA from 3′ BLV end (15). The interplay between proviral BLV DNA, BLV microRNAs, and leukoses remains unclear. There is an evidence for counteraction between proviral BLV DNA expression and microRNA expression as a result of host immune system selection pressure against cellular clones expressing BLV proteins.

In our previous studies, we demonstrated that cows, which are infected with BLV virus, have lower *NK-lysin* gene expression (that is the functional marker of T and NK killers) and have an increased thrombocyte count as compared to uninfected cows (16). It could indicate that the major BLV-induced infection event is the suppression of the host immune system. This would explain why BLV vaccination has such a low response rate (17). It is important to know if there is a connection between proviral BLV DNA expression, BLV microRNA expressions, leukoses, and the suppression of the host immune system, because BLV microRNA participates in pathogenesis induced by this retrovirus (14). The key proteins participating in maturation of microRNA transcript, as well as microRNA BLV transcript, are Dicer (*dc1*) and Argonaute (*ago2*). Therefore, in this work, we evaluate correlations between leukocytosis, expression of reverse transcriptase BLV *pol, NK-lysin* (that marks the activity of T and NK killers), BLV microRNA, genes *dc1* and *ago2* (their products participate in microRNA maturation), as well as the gene encoding cellular receptor for BLV (*blvr*).

## Materials and Methods

Black-and-white Holsteinized cows, age between 2 and 5 years from ZAO Mozhayskoe farm (57 animals), were included in this study. The blood was taken from caudal veins of the animals. All animal care and experimental treatments were performed in compliance with the rules of and regulations of the Ministry of Agriculture of Russian Federation (directive#183, 04.16.2013 and directive#56, 02.16.2016).

Erythrocytic and lymphocytic profiles, as well as erythrocytes' properties, were measured using automatic hematological analyzer Abacus junior Vet5 (≪Diatron≫, Austria; the working principle is based on Coulter's method). The fresh peripherical samples of each animal's blood (4 ml), stabilized with EDTA, were used. The animals, infected with proviral DNA BLV, were found using radial immunodiffusion Mancini approach (RID) and our own PCR method (18).

DNA was isolated from 100 μl of whole blood with a reagent kit for DNA isolation from clinical samples “M-sorb” (Sintol, Russia) following the manufacturer's recommendations. The tested animals were distributed into two groups: those carrying integral BLV proviral DNA and infection-free. The total RNA was isolated from 1 ml of cow whole blood with a reagent ExtractRNA (Eurogen, Russia) following the manufacturer's recommendations. The RNA was processed with DNAse I (≪Thermo Fisher Scientific≫, USA), a first cDNA strand was synthesized with an MMLV RT kit by Eurogen (Russia) following the manufacturers' recommendations.

Comparative gene expression analysis (*NK-lysin, dc* and *ago*) was performed by quantitative techniques of real-time polymerase chain reaction with an intercalating dye SYBR Green and ready-to-use mix qPCRmix-HS SYBR (Eurogen, Russia) and a LightCycler 96 (≪Roche≫, Switzerland). The gene of a ribosomal protein RPLPO was used as a reference. Amplification was performed in 20 mcl of a reaction mix containing direct and reverse primers (0.2 mcM each) and c DNA (2 mcl). The following specific primers were used: *NK-lysin -* 5′-CCTCGGTGCTCCTGGTYGC−3′, 5′-GGTCACCCTGGGGATCCTC−3′; *pol -* 5′–GCAGGCCGATATAACCCAT−3′, 5′–TGCTGGCAAAACCTGACAAAG−3′; *dicer1* – 5′-GAGTCACCGTGGAAGTGGTC-3′, 5′-CTCTCAAACCGCATCCCTCT-3′; *ago2* – 5′- GGCAGGACAGAGATGCATAA-3′, 5′-GCAGCAGGATGTTGTTCACG-3′; *blvr*- 5′-CTATCGGACCAGTATGTGAAG−3′, 5′-CTCCTCGGTGACGATGTCC−3′ 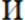 *RPLPO*- 5′-CAACCCTGAAGTGCTTGACAT−3′, 5′-CAGATGGATCAGCCAAGAAG−3′.

PCR was performed with LightCycler 96 (≪Roche≫, Switzerland) under the following conditions: denaturation: 95°C, 15 s, primers annealing: 60°C, 15 s, elongation: 72°C, 15 s, 40 cycles. Fluorescence signal was picked up by a SYBR channel. Specificity of the reaction was tested with the curves of temperature dissociation of the resulting amplicons.

To determine BLV microRNA, total RNA was isolated from 1 ml of whole cow blood with ExtractRNA (Eurogen, Russia), following the manufacturer's recommendations. RNA was processed with DNA-se I (≪Thermo Fisher Scientific≫, USA); a first cDNA strand was synthesized with MMLV RT kit (Eurogen, Russia), following the manufacturers' recommendations. Expression of B4 microDNA was analyzed by real-time PCR with 5X ready-to-use mixture qPCRmix-HS (Eurogen, Russia), primers B4- F1: 5′-GGAAAGAACTAACGCTGACG-3′ and B4- R1: 5′-AGGGCGTAAAAAGCGGAAGC-3′, probes TaqMan miR-B4-5p: 5′-(FAM) AAGCGAGAGGCTCTGGTGCTGG-BHQ-1 and miR-B4-3p: 5′-(HEX)TAGCACCACAGTCTCTGCGCCTTT-3′ - BHQ2. PCR was performed in LightCycler 96 (≪Roche≫, Switzerland) at the following conditions: denaturation: 95°C, 10 s, primers annealing: 58°C, 10 s, elongation: 72°C, 15 s, 40 cycles. Fluorescence signal was picked up by FAM and HEX channels. Specificity of PCR was tested by sequencing of resulting 149 b.p.-long amplicons by Sanger.

Gene expression quantification was performed using LightCycler 96 SW1.1 (≪Roche≫, Switzerland) software. Statistical data analysis was implemented with Statistica 6.0 software (≪StatSoft Inc.≫, USA). Differences for Pearson's correlation coefficients were considered being statistically significant for *P* < 0.05. The tables present means (X) and standard deviation (x).

## Results and Discussion

Based on the results of the presence of antibodies (RID+), pro-viral DNA in a genome (BLV+), and leukocytosis, all animals were divided into three groups. The first included the animals without Infection (RID- and without pro-viral DNA BLV – BLV−) in the blood, **Control** in what follows). The second included infected animals (RID+ and with pro-viral DNA BLV – BLV+) with relatively **L**ow level of **L**eucocytes, between 6.4 and 17.7 × 10^9^/L (**RID+** BLV+**_LL**, in what follows). The third included infected animals (RID+ BLV+) with very **H**igh **L**eucocyte count, more than 18 × 10^9^/L (**RID+** BLV+**_HL** in what follows). Independently, we also measured BLV-miR-B4 expression in all three groups. Table 1 presents estimated abundance of different leukocyte populations for all three groups. Table 2 presents expression of the genes of pro-viral DNA BLV (gene *pol*), microRNA BLV (BLV-miR-B4), and genes of microRNA maturation (*dc1* 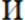 *ago2)* in four groups. For Table 2, we considered all cows with microRNA expression as a separate group, without paying attention to the number of leukocytes. All these cows (9 animals) were RID+ and with pro-viral DNA BLV (**RID+** BLV+**_miR-B4** column in Table 2).

**Table 1:** Comparative analysis of erythrocyte and leucocyte profiles in peripheral blood of three groups of animals (**Control, RID+** BLV+**_LL** and RID+ BLV+_HL) in the cows of ZAO Mozhayskoe farm (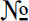
1).

**Cell populations**	**RID+BLV+_LL**		**Control**		**RID+BLV+_HL**	
	***N***	**erythrocytes - × 10^**12**^, leukocytes - × 10^**9**^/l**	***N***	**erythrocytes - × 10^**12**^, leukocytes - × 10^**9**^/l**	***N***	**erythrocytes - × 10^**12**^, leukocytes - × 10^**9**^/l**
Erythrocytes	16	6.15 ± 0.17	7	6.86 ± 0.22	6	6.34 ± 0.36
Leukocytes	16	11.73 ± 0.88	7	10.21 ± 0.70^**^	6	25.49 ± 1.01^**^
Lymphocytes	16	6.12 ± 0.73	7	4.85 ± 0.48^***^	6	21.70 ± 1.08^***^
Monocytes	16	0.28 ± 0.09	7	0.52 ± 0.15^*^	6	1.61 ± 0.24^*^
Neutrophils	16	4.63 ± 0.83^*^	7	4.54 ± 0.50^*^	6	1.66 ± 0.35^*^
Eosinophils	16	0.59 ± 0.09	7	0.45 ± 0.16	6	0.51 ± 0.18
Basophils	16	0.0088 ± 0.0027	7	0.0114 ± 0.0014^*^	6	0.0067 ± 0.0021^*^
Thrombocytes	16	160.81 ± 47.55	7	12.90 ± 9.91^*^	6	122.33 ± 78.28^*^
Mean corpuscular volume	16	46.12 ± 0.86	7	44.86 ± 2.11	6	46.50 ± 1.09
Erythrocytes variability with regards to their diameter, %	16	18.55 ± 0.34	7	20.83 ± 0.52	6	18.83 ± 0.31

N, numbers of animals;

* *P < 0.05*,

** *P < 0.01*,

*** *P < 0.001*.

**Table 2:** Expression of genes *NK-lysin, blvr, dc1* and *ago2* in peripheral blood of four groups of animals (**Control, RID+BLV+_LL, RID+BLV+_HL** and animals with BLV-miR-B4 expression, **RID+BLV+_miR-B4**) in the cows of ZAO Mozhayskoe farm (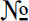
1).

**Gene expression**	**RID+BLV+_miR-B4**		**RID+BLV+_LL**		**Control**		**RID+BLV+_HL**	
	***N***	**Leukocytes**	***N***	**Leukocytes**	***N***	**Leukocytes**	***N***	**Leukocytes**
		18.39 ± 2.29^**^		11.73 ± 0.88		10.21 ± 0.70		25.49 ± 1.01^**^
*NK-lysin*	9	38.32 ± 7.43^*^	16	54.49 ± 8.98	20	78.01 ± 14.32^*^	6	36.15 ± 11.04^*^
*pol*	9	2.72 ± 1.38	14	Absent (2, 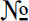 197 and 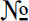 7,575) have expression of *pol*. and excluded from the analysis	20	Absent	6	3.9 ± 3.6 (from 1.2 to 13.3 conditional units)
*Blvr*	9	9.88 ± 1.93	15	11.26 ± 2.17	7	7.10 ± 1.50	6	12.18 ± 2.37
*BLV-miR-B4*	9	9	16	5 from 16 (31%)	20	Absent	6	4 from 6 (67%)
*Dc1*	9	22.88 ± 12.34^*^	14	4.35 ± 1.55	20	2.03 ± 0.56^*^	6	38.09 ± 16.16^*^
*Ago2*	9	11.79 ± 2.75^*^	14	7.08 ± 1.33	20	4.54 ± 0.89^*^	6	16.82 ± 3.58^*^

N, amount of animals;

* *P < 0.05*,

** *P < 0.01*,

***P < 0.001.

Agranulocyte and granulocyte components of the leucocyte populations (except eosinophil population) were significantly different between control and BLV-infected groups only for **RID+** BLV+**_HL** and **RID+** BLV+**_miR-B4** groups (Tables 1, 2). There were no significant differences for erythrocyte component between groups (Table 1).

There were no correlation between *pol* expression and leukocytosis. Among the animals with relatively low leukocyte count (**RID+** BLV+**_LL**, 16 cows), there were only two animals with detectable *pol* expression*. One* of them had relatively low number of leukocytes *(*7.83 × 10^9^/L) and another one relatively high (17.76 × 10^9^/L). Among the animals with high leukocytosis, the maximum expression of *pol* gene (13.3 relative expression units) was found for the animal with the average leukocytosis for this group (24.9 × 10^9^/L). Leukocytosis also did not correlate with microRNA expression. The expression was observed for only 9 out of 22 animals (**RID+** BLV+**_LL, RID+** BLV+**_HL** groups), with overall highly variable number of leukocytes.

Statistically significant differences in *NK-lysin, dc1* and *ago2* gene expressions were found only between **Control** group and **RID+** BLV+**_HL, RID+** BLV+**_miR-B4** groups (Table 2).

Expression of *NK-lysin, dc1* and *ago2* genes in **RID+** BLV+**_miR-B4** and **RID+** BLV+**_HL** groups were very similar (*NK-lysin* 38.32 ± 7.43; *dc1* 22.88 ± 12.34; *ago2* 11.79 ± 2.75, respectively). As compared to control, the group with microRNA expression was characterized by underexpression of *NK-lysin* 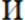 and overexpression of enzymes, central for microRNA maturation.

In Tables 3–**6**, we present correlations between various leukocyte populations and gene expression. For **Control** group (Table 3), there were positive correlations between leukocytes and neutrophils. There were no correlations between different cell populations and *dc1* 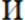 *ago2* expression, while gene expressions themselves were significantly correlated (*P* < 0.5).

**Table 3:** Correlations between the number of various agranulocytes, granulocytes, and *NK-lysin, dc1* and *ago2* expressions in the peripheral blood of infection free cows (RID- and without inserted proviral DNA, **Control** group) in the cows of ZAO Mozhayskoe farm.

**Parameters**	**Leukocytes**	**Lymphocytes**	**Monocytes**	**Neutrophils**	**Eosinophils**	**Basophils**	**Thrombocytes**	***dc***	***Nklys***	***ago***
Leukocytes	1.00									
Lymphocytes	0.71^*^	1.00								
Monocytes	0.02	0.47^*^	1.00							
Neutrophils	0.85^*^	0.27	−0.42	1.00						
Eosinophils	0.22	0.24	0.65^*^	−0.03	1.00					
Basophils	0.57^*^	0.42	0.03	0.42	0.38	1.00				
Thrombocytes	−0.18	0.02	0.49^*^	−0.36	0.57^*^	−0.09	1.00			
*dc*	−0.03	0.06	−0.01	−0.09	0.15	0.37	0.18	1.00		
*NKlys*	−0.46^*^	−0.52^*^	−0.21	−0.24	−0.15	−0.28	0.04	0.09	1.00	
*ago*	0.16	0.17	0.09	0.07	0.22	0.43	0.13	0.52^*^	0.25	1.00

* *P < 0.05*.

Interestingly, *NKlys* gene expression was negatively correlated with the number of lymphocytes and leukocytes. That is, the higher was the number of leukocytes and lymphocytes, the lower was *NKlys* expression, the marker of *T* and NK-killer cells. It could indicate that in this particular group of animals, immune effector cells were relatively inactive, leading to the increased number of lymphocytes and leukocytes, even without BLV infection (Table 3).

Overall, for the group of infected animals with low leukocyte count (**RID+** BLV+**_LL**), there were not as many significant correlations as in **Control** group. There were no correlations between *dc* and *ago2* gene expressions, as well as *NK-lysin* gene expression and the number of lymphocytes and leukocytes (Table 4). Interestingly, for the group of infected animals with high leukocyte numbers (RID+ BLV+_HL), *dc1* 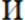 *ago2* gene expressions were also non-correlated (Table 5).

**Table 4:** Correlations between the number of various agranulocytes, granulocytes and *NK-lysin, dc1* and *ago2* expressions in the peripheral blood of infected cows with low leukocyte count (**RID+BLV+_LL** group).

**Parameters**	**Leukocytes**	**Lymphocytes**	**Monocytes**	**Neutrophils**	**Eosinophils**	**Basophils**	**Thrombocytes**	***dc***	***Nklys***	***ago***
Leukocytes	1.00									
Lymphocytes	0.59^*^	1.00								
Monocytes	0.31	0.60^*^	1.00							
Neutrophils	0.57^*^	−0.32	−0.31	1.00						
Eosinophils	−0.02	0.47	0.21	−0.54^*^	1.00					
Basophils	0.001	−0.25	−0.13	0.20	0.26	1.00				
Thrombocytes	−0.03	0.46	0.44	−0.52^*^	0.22	−0.66^*^	1.00			
*dc*	−0.15	−0.32	−0.35	0.17	−0.40	−0.17	0.14	1.00		
*NKlys*	−0.23	−0.27	−0.31	−0.01	−0.16	−0.15	−0.21	0.17	1.00	
*ago*	−0.04	−0.39	−0.29	0.34	−0.39	0.36	−0.63^*^	0.09	0.47	1.00

* *P < 0.05*.

**Table 5:** Correlations between the number of various agranulocytes, granulocytes and *NK-lysin, dc1* and *ago2* expressions in the peripheral blood of infected cows with high leukocyte count (**RID+BLV+_HL** group).

**Parameters**	**Leukocytes**	**Lymphocytes**	**Monocytes**	**Neutrophils**	**Eosinophils**	**Basophils**	**Thrombocytes**	***pol***	***dc***	***Nklys***	***ago***
Leukocytes	1.00										
Lymphocytes	0.87^*^	1.00									
Monocytes	−0.12	−0.45	1.00								
Neutrophils	0.30	−0.20	0.63	1.00							
Eosinophils	−0.08	−0.14	−0.55	0.08	1.00						
Basophils	−0.57	−0.87^*^	0.54	0.51	0.30	1.00					
Thrombocytes	0.11	0.29	−0.80	−0.37	0.69	−0.34	1.00				
*pol*	−0.02	−0.02	−0.66	−0.02	0.92^*^	0.17	0.54	1.00			
*Dc1*	−0.01	−0.20	−0.32	0.36	0.86^*^	0.44	0.29	0.91^*^	1.00		
*NKlys*	0.03	−0.27	0.45	0.61	−0.005	0.25	0.05	−0.29	−0.04	1.00	
*Ago2*	0.24	−0.10	0.19	0.73	0.25	0.33	−0.31	0.40	0.68	0.19	1.00

* *P < 0.05*.

The least amount of significant correlations was observed for the group of infected animals with high leukocyte count (**RID+** BLV+**_HL**). Still, there was positive correlation between the number of leukocytes and lymphocytes and one new positive correlation—between *pol* and *dc1* gene expressions (Table 5).

Interestingly, for 3 out of 9 infected animals with miRNA expression (group **RID+** BLV+**_miR-B4)**, we did not observe *pol* expression, despite the insertion of pro-viral DNA BLV (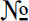

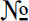
 7,687, 384, 7,531).

Table 6 presents the results of correlation analysis for nine infected animals with miRNA expression (group **RID+** BLV+**_miR-B4)**.

**Table 6:** Correlations between the number of various agranulocytes, granulocytes and *NK-lysin, dc1*, and *ago2* expressions in the peripheral blood of infected cows with microRNA expression (RID+BLV+, with inserted BLV proviral DNA and microRNA expression, group **RID+BLV+_miR-B4**.

**Parameters**	**Leukocytes**	**Lymphocytes**	**Monocytes**	**Neutrophils**	**Eosinophils**	**Basophils**	***pol***	***Dc1***	***NKlys***	***Ago2***	***Blvr***
Leukocytes	1.00										
Lymphocytes	0.90^*^	1.00									
Monocytes	0.72^*^	0.77^*^	1.00								
Neutrophils	−0.20	−0.61	−0.46	1.00							
Eosinophils	0.06	0.23	−0.18	−0.41	1.00						
Basophils	−0.03	−0.18	0.03	0.31	−0.15	1.00					
*pol*	0.46	0.50	0.22	−0.31	0.67^*^	−0.01	1.00				
*Dc1*	0.53	0.51	0.39	−0.22	0.53	0.17	0.93^*^	1.00			
*NKlys*	0.28	0.21	0.43	−0.01	−0.13	0.03	−0.08	0.02	1.00		
*Ago2*	0.55	0.47	0.66	−0.14	0.19	0.26	0.58	0.82^*^	0.39	1.00	
*Blvr*	0.43	0.55	0.46	−0.50	0.67^*^	0.05	0.81^*^	0.89^*^	0.18	0.82^*^	1.00

* *P < 0.05*.

For this group, statistically significant correlations were observed mostly between *pol, dc1* and *blvr*, between *dc1, blvr* and *Ago2, dc1*, and *Ago2* gene expression, besides correlation between the number of leukocytes and lymphocytes (Table 6).

In what follows, we briefly discuss the obtained data.

Significant differences in leukocyte populations (agranulocytes, neutrophils, thrombocytes) were observed only between control and infected animals with high leukocyte count (RID+ BLV+_HL group, Table 1). The same trend was observed for basophils and eosinophils; however, their frequencies were low and they were not included in the further analysis.

For two groups of infected animals with different leukocyte counts (RID+ BLV+_HL and RID+ BLV+_LL), expression of BLV microRNA and *pol* gene was not always correlated. There were animals with and without synchronized expression. The postulated exclusiveness, either proviral BLV DNA transcription or BLV microRNA expression, was not observed *in vivo* (Table 2). However, the leukocyte counts and *Nklys, dc1* and *ago2* gene expressions were significantly different between control, infected animals with low leukocyte count on the one hand, and infected animals with high leukocyte count infected animals with BLV microRNA expression on the other hand (Table 2). All groups are not statistically different based on *blvr* gene expression.

For control group, we found significant positive correlations between the number of leukocytes, lymphocytes, and neutrophils, negative correlations between the number of leukocytes, lymphocytes, and *Nklys* gene expression, and positive correlations between expressions of two key enzymes for microRNA maturation - *dc1* and *ago2* (Table 3). That is, the number of cells for adaptive (lymphocytes, leukocytes) and innate (neutrophils) immune responses are positively correlated, the increase of adaptive immune response leads to decrease *Nklys* gene expression, and key enzymes for microRNA maturation are co-expressed in control group.

For infected animals with low number of leukocytes, we found significant positive correlations between the number of leukocytes, lymphocytes, monocytes, and neutrophils (Table 4). There were no correlations between expressions of two key enzymes for microRNA maturation. *Pol* gene was expressed in only 2 out of 16 cows in this group; therefore, correlations for this gene were not available (Table 2).

For infected animals with high number of leukocytes, we found significant positive correlations between the number of leukocytes and lymphocytes, as well as between expression of genes *pol* and *dc1* (Table 6). Unlike in control group, in this group of infected animals, there was no significant correlation between *dc1* and *ago2* gene expressions (Tables 3, 6).

For infected animals with BLV microRNA expression, we found significant positive correlations between the numbers of leukocytes, lymphocytes, monocytes *pol* and *dc1, dc1*, and *ago2* gene expressions (Table 6). Interestingly, there were also correlations between *blvr, pol, dc1*, and *ago2* gene expressions (Table 6). In our previous studies, *blvr* gene expression was also increased in BLV infected animals; however, its expression didn't correlate with other features (16). Generally, *blvr* is highly expressed in juvenile lymphocytes and its expression gradually decreases during terminal differentiation (19).

## Conclusion

In conclusion, the data presented here, particularly the apparent absence of BLV DNA expression in pro-leucosis and leucosis cells, where BLV microRNA is expressed, suggest that the BLV infection targets juvenile lymphocytes. In the process of maturation, those lymphocytes are further divided into two groups. One group expresses BLV DNA and another one expresses BLV microRNA that decreases host immune response against cells, expressing BLV proteins.

## Data Availability Statement

The datasets generated for this study are available on request to the corresponding author.

## Ethics Statement

The animal study was reviewed and approved by Ministry of Agriculture of Russian Federation (directive#183, 04.16.2013 and directive#56, 02.16.2016).

## Author Contributions

VG designed the study, administered the oversight of the project, and wrote the original draft. TG and GK performed the sample collection and experiments described. GG and BZ translated the manuscript to English, performed editing, correction, and led the results interpretation and discussion.

## Conflict of Interest

The authors declare that the research was conducted in the absence of any commercial or financial relationships that could be construed as a potential conflict of interest.
